# The potential role of dietary patterns in modifying the association between ambient PM_2.5_ exposure and mortality in elderly Hong Kong Chinese

**DOI:** 10.1186/s12940-026-01296-6

**Published:** 2026-04-17

**Authors:** Shu-Yi Li, Jason Leung, Zhi-Hui Lu, Kin-Fai Ho, Yi Su, Blanche Yu, Timothy Kwok

**Affiliations:** 1 Department of Medicine and Therapeutics, Prince of Wales Hospital, The Chinese University of Hong Kong, Shatin, Hong Kong, China; 2 Jockey Club Centre for Osteoporosis Care and Control, The Chinese University of Hong Kong, Hong Kong, China; 3 School of Nursing, The Hong Kong Polytechnic University, Hong Kong, China; 4 The Jockey Club School of Public Health and Primary Care, The Chinese University of Hong Kong, Hong Kong, China; 5 Department of Epidemiology and Biostatistics, Key Laboratory of Molecular Epidemiology of Hunan Province, School of Public Health, Hunan Normal University, Changsha, China; 6 Faculty and Planning Office, Faculty of Medicine, The Chinese University of Hong Kong, Hong Kong, China

**Keywords:** Dietary pattern, Air pollution, Fine particulate matter, Mortality, Older adults, Prospective cohort

## Abstract

**Background:**

The potential of dietary patterns to modify the associations between long-term ambient fine particulate matter (PM_2.5_) exposure and all-cause, respiratory, and circulatory mortality remains unclear.

**Methods:**

A total of 3,937 older adults (≥ 65 years) living in the community were enrolled in a prospective cohort study from 2001 to 2003 in Hong Kong. Assessment of the Diet Quality Index-International (DQI-I), Dietary Inflammatory Index (DII), the Mediterranean-DASH Intervention for Neurodegenerative Delay (MIND) diet was carried out utilizing a 280-item food frequency questionnaire. Annual PM_2.5_ concentrations were estimated via land use regression models. Mortality outcomes were sourced from official death registry. Time-varying Cox proportional hazards models were applied to estimate hazard ratios (HRs) and their 95% confidence intervals (CIs).

**Results:**

During a median of 16.8 years follow-up, 1,856 deaths were recorded. PM_2.5_ exposure significantly increased the risks of all-cause mortality, respiratory and circulatory mortality. The MIND diet interacted with PM_2.5_ exposure on all-cause (*p*-interaction = 0.008) and respiratory mortality (*p*-interaction = 0.022). Higher MIND diet scores (≥ median) attenuated the adverse PM_2.5_ exposure-mortality association, showing lower risks for all-cause (HR: 1.18, 95% CI: 1.06–1.32) and respiratory mortality (HR: 1.14, 95% CI: 0.91–1.41). In contrast, those with lower MIND scores (< median) had elevated mortality risks, with HRs of 1.45 (95% CI: 1.27–1.65) for all-cause mortality and 1.60 (95% CI: 1.24–2.06) for respiratory mortality. No significant interaction was observed for circulatory mortality. Besides, no interaction was found between PM_2.5_ exposure with DQI-I or DII in relation to mortality.

**Conclusions:**

Adherence to MIND diet may mitigate the detrimental effects of long-term exposure to PM_2.5_ on all-cause and respiratory mortality in older adults, whereas overall diet quality or an anti-inflammatory diet showed no protective effects.

**Supplementary Information:**

The online version contains supplementary material available at 10.1186/s12940-026-01296-6.

## Background

Air pollution has increasingly been recognized as a significant environmental and public health issue [1]. Exposure to ambient air pollution is a major contributor to mortality, with an estimated 4.5 million premature deaths worldwide in 2019 [2]. Fine particulate matter (PM_2.5_) is especially hazardous to health due to its ability to reach deep into the respiratory system, entry the circulatory system, and disseminate throughout the body, thereby contributing to disease onset and progression and increasing mortality risk [3]. Accumulating number of studies have observed the detrimental effects of PM_2.5_ exposure on mortality risk [4]. While policy decisions and societal efforts to improve air quality and reduce emissions are essential, individuals must also take proactive steps to protect themselves from the adverse health consequences of air pollution [5].

Emerging evidence suggests that lifestyle factors, including diet, may modify the susceptibility of individuals to the adverse health effects of ambient air pollution [6–9]. For example, a prospective cohort study using the UK biobank data found that high vegetable intake attenuated the adverse effects of ambient air pollution on overall mortality risk, although no significant interaction was found for cardiovascular disease (CVD) mortality [10]. Similarly, in a large prospective U.S. cohort, individuals with higher Mediterranean diet scores had lower CVD mortality risk linked to long-term ambient PM_2.5_ exposure [11]. However, findings remain inconsistent, as no interaction was found between lifestyle factors and PM_2.5_ exposure on overall mortality risk in the UK biobank study [12] and no modification of diet was found in the association between air pollution and incident stroke in U.S. men [13]. Both PM_2.5_ exposure levels and dietary habits may vary across different regions. Few studies have investigated the potential role of diet in modifying the adverse effects of air pollution on mortality risk in Asian populations, especially in older adults [10, 11], and further studies across diverse populations are needed.

Impaired redox and inflammatory pathways are important mechanisms through which PM_2.5_ exposure induces both local and systemic responses [14, 15]. Previous studies suggest that dietary patterns may counteract the air pollutant-triggered oxidative damage and pro-inflammatory responses, thereby biologically interacting with the adverse impacts of air pollutants on health outcomes [16, 17]. However, it remains unclear whether specific anti-inflammatory diets, antioxidant-rich dietary patterns, or overall diet quality can attenuate the detrimental effects of PM_2.5_ exposure on mortality. To address this, the present study explored three dietary indices that capture different aspects of diets. The Dietary Inflammatory Index (DII) was a validated tool to quantify the inflammatory potential of dietary intake, with lower scores reflecting diets associated with anti-inflammatory effects [18]. The Mediterranean-Dietary Approach to Systolic Hypertension (DASH) Intervention for Neurodegenerative Delay (MIND) diet is based on the DASH and Mediterranean diets, incorporating neuroprotective foods such as green leafy vegetables and berries, which possess antioxidant and anti-inflammatory properties [19]. Besides, the Diet Quality Index-International (DQI-I) provides a holistic evaluation of overall dietary quality across four key dimensions: food variety, dietary adequacy, nutrient moderation, and overall balance [20]. While lower DII scores (anti-inflammatory diets) and greater adherence to MIND diet may mitigate inflammation and oxidative stress to reduce mortality risk [21, 22], DQI-I captures broader dietary patterns that may influence health through mechanisms beyond inflammation and oxidative stress. For instance, our previous study found that higher DQI-I scores reduced overall mortality risk in women, independent of inflammatory markers [23]. By comparing these three dietary indices, the study aimed to examine the protective effects of diet against PM_2.5_-related mortality from anti-inflammatory/antioxidant diets or overall dietary quality.

Therefore, this study aimed to examine the associations between long-term ambient PM_2.5_ exposure and the risk of all-cause, respiratory, and circulatory mortality over a 16-year of follow-up period among community-dwelling older adults in high-density urban areas, Hong Kong. In addition, we examined the potential modifying effects of DQI-I, DII and MIND diet scores on these associations. Stratified and joint analyses were conducted to assess the associations between these dietary patterns and ambient PM_2.5_ exposure in relation to mortality risk. While reducing ambient PM_2.5_ levels requires long-term policy interventions, identifying protective dietary patterns may offer an actionable strategy for vulnerable elderly populations to mitigate the health risks of living in polluted environments.

## Methods

### Study participants

Data were obtained from the Mr. OS and Ms. OS study [24, 25]. A prospective cohort study recruited 2,000 men and 2,000 women aged over 65 years between 2001 and 2003 in Hong Kong. An equal number of participants was selected from three age groups (65 to 69, 70 to 74, more than 75 years) using a stratified sampling method. By June 2020, participants were followed up for four rounds during the periods 2003–2005, 2005–2007, 2008–2010, and 2015–2017. Participants were excluded if they had missing data on baseline residential address (*n* = 45), dietary data (*n* = 5), or they reported implausible energy intakes (> 3500 kcal/d or < 500 kcal/d for women; > 4000 kcal/d or < 800 kcal/d for men; *n* = 13) [26]. The final sample was 3,937 participants in the present analysis.

### PM_2.5_ concentrations

Annual PM_2.5_ concentrations were estimated using land use regression (LUR) models based on the baseline residential addresses of each participant. Detailed methodology of LUR models has been previously published elsewhere [27]. Briefly, the LUR models was developed specifically for the complex high-density urban environment of Hong Kong. The model integrated multiple spatial-temporal predictors, including high-resolution PM_2.5_ monitoring data of 5-year long-term hourly monitoring data (2011–2015) from 15 local air quality monitoring network (AQMN) stations of the Hong Kong Environmental Protection Department (HKEPD), traffic network density and volume, urban land use, population density and geographic coordinates of monitoring sites, and meteorological variables (including ambient temperature, relative humidity levels, wind speed and direction) obtained from the Hong Kong Observatory, and urban morphological parameters derived from a 2-meter resolution digital surface model (DSM) to capture the building geometry and wind availability in Hong Kong. Validation of this LUR model showed an adjusted 𝑅^2^ of 0.671 and a leave-one-out cross-validation root-mean-square error (LOOCV RMSE) of 2.612 µg/m^3^ for estimating annual PM_2.5_ concentration. Ambient PM_2.5_ exposure was treated as a time-varying variable and computed as the average concentration ranging from the calendar year prior to the previous survey wave up to the calendar year prior to the subsequent survey wave (or the year prior to death). Specifically, the exposure assigned to the follow-up period between the baseline and the first survey wave was the average of annual PM_2.5_ concentrations from the year before the baseline to the year before the first follow-up; similarly, exposure for subsequent intervals was updated as the average from the year prior to the preceding wave to the year prior to the current wave or death. PM_2.5_ concentrations were categorized into tertiles: lower exposure (first tertile, 22.31–31.52 µg/m^3^), medium exposure (second tertile, 31.53–34.51 µg/m^3^), higher exposure (third tertile, 34.52–41.80 µg/m^3^).

### Dietary patterns

A 280-item food frequency questionnaire (FFQ) was used at baseline to estimate dietary intake over the prior year [28]. Information on the frequency and portion sizes of food consumed was collected via face-to-face interviews. A catalog of food portion pictures was used to aid in estimating food portion sizes. The daily intake of nutrients and energy were derived using information from the Chinese Food Composition Table [29] and McCance and Widdowson [30].

#### Diet Quality Index-International (DQI-I)

DQI-I was used to estimate overall dietary quality [20]. It evaluates four key components of diet: food variety, dietary adequacy, nutrient moderation, and overall balance. A total DQI-I score ranging from 0 to 100 was calculated, where higher values indicate better overall diet quality. As empty-calorie foods could not be evaluated in the moderation component, the maximum achievable moderation score was limited to 24, reducing the total possible DQI-I score to 94 rather than 100.

#### Dietary Inflammatory Index (DII)

DII was developed to quantify assess the overall inflammatory potential of dietary intake, based on a comprehensive literature review of 45 food parameters and their effects on inflammatory biomarkers [18]. The detailed algorithms of DII calculation have been published elsewhere [18, 31, 32]. In this study, 30 food parameters were included in the computation. DII scores ranged from −4.66 to 5.13, with lower and negative scores indicating greater anti-inflammatory potential, and higher and positive scores reflecting greater pro-inflammatory potential of the diet.

#### Mediterranean-DASH Intervention for Neurodegenerative Delay (MIND) Diet

The MIND diet combines the principles of the Mediterranean and DASH diets and incorporates modifications based on evidence to emphasize neuroprotective dietary components [19]. The MIND diet includes ten food groups beneficial for brain health: whole grains, green leafy vegetables, other vegetables, berries, fish and seafood, poultry, nuts, beans, olive oil, and wine. It also includes five food groups considered unhealthy: red meat and its products, deep-fried fast foods, sweets and pastries, butter and cheese [19]. Adherence scoring assigns ordinal values (0, 0.5, 1) to weekly intake frequencies for all components except olive oil. Olive oil was used as the primary household oil was scored as 1, with all other cases scored as 0. The maximum possible MIND diet score was 15, with higher scores indicating greater dietary adherence.

Participants were categorized into binary groups based on sex-specific median values for each dietary index. For the DQI-I, scores of ≥ 64 in men and ≥ 66 in women were considered indicative of higher diet quality, while lower values signified lower diet quality. MIND diet adherence was similarly dichotomized at the sex-specific median, defined as ≥ 4.5 for both men and women. For the DII, diets were classified as anti-inflammatory when scores were < −0.82 in men and < −0.21 in women; scores at or above these medians were considered pro-inflammatory.

### Mortality ascertainment

Mortality outcome for each participant was sourced from the Hong Kong Government Death Registry Database and were identified according to the ICD-10 codes. The three primary endpoints in this study included all-cause mortality, respiratory mortality (ICD-10: J00-J99), and circulatory mortality (ICD-99: I00-I99).

### Covariate assessments

Covariates included in this study were measured at baseline and follow-up visits. Demographic characteristics (including sex, age, educational attainment, and marital status), lifestyle behaviors (such as smoking status and alcohol consumption), and medical history were obtained using a standardized questionnaire [32]. Medical history included 12 types of chronic diseases or symptoms such as diabetes, hypertension, CVD, stroke, chronic obstructive lung disease (COPD), cancer, and so on [32]. The total number of chronic conditions reported by each participant was grouped into three categories: no chronic diseases; 1 to 2; 3 or more. Physical activity levels were assessed using the 12-item Physical Activity Scale of the Elderly (PASE) [33]. This questionnaire estimated the average daily duration of engagement in leisure, household, and occupational activities over a one-week period. Body weight was measured using the Physician Beam Balance Scale and height was measured with the Holtain Harpenden stadiometer. Body mass index (BMI) was then calculated and classified into four groups: less than 18.5 kg/m^2^; 18.5 to 22.9 kg/m^2^; 23 to 27.4 kg/m^2^; and 27.5 kg/m^2^ or more [34].

### Statistical analysis

Participant characteristics at baseline were described using means and standard deviations (SDs) for continuous variables, and proportions (%) for categorical variables.

To examine associations between long-term ambient PM_2.5_ exposure and the risks of all-cause, respiratory and circulatory mortality, time-varying Cox proportional hazards model was used. Cox models used follow-up time as the time scale. The follow-up period was calculated as the time between consecutive survey waves, or the last available survey wave until death or the end of mortality follow-up (June 2020). PM_2.5_ exposure and covariates, including age, marital status, smoking status, current alcohol drinking, physical activity level, number of chronic diseases, and BMI categories, were time-varying variables. Sex and educational level were time-constant variables. Associations were expressed as hazard ratios (HRs) with 95% confidence intervals (CIs) across PM_2.5_ tertiles and per 5 µg/m³ increase in exposure. Sex (male; female) and age (continuous) were adjusted in Model (1). Education level (no education; primary or below; secondary or above), marital status (married, widowed, single/separated/divorced), smoking (never, former, current), alcohol drinking (yes; no), number of chronic diseases (0; 1–2; ≥3 diseases), physical activity (continuous), BMI categories (< 18.5; 18.5–22.9; 23-27.4; ≥27.5 kg/m^2^) and total energy intake (continuous) were additionally adjusted in Model (2). Furthermore, to examine potential exposure-response association between PM_2.5_ exposure and mortality risk, restricted cubic spline (RCS) models were used. We evaluated models with 3, 4, 5 knots and selected 3 knots for the final models, as they had the lowest Akaike Information Criterion (AIC) and Bayesian Information Criterion (BIC) values across the outcomes [35]. The knots were located at the default 10th, 50th, and 90th percentiles of the PM_2.5_ exposure distribution, with the 1st percentile set as the reference point. In addition, the population distribution of PM_2.5_ concentrations was plotted as histograms and jointly presented with the RCS curves.

Stratified analyses were conducted to examine whether dietary patterns modified the association between ambient PM_2.5_ exposure and mortality risk. Subgroup analysis defined by dietary pattern scores (< vs. ≥ sex-specific median) was conducted. The interactions between dietary pattern scores and PM_2.5_ exposure in relation to mortality risk were estimated by incorporating multiplicative interaction terms into time-varying Cox proportional hazards models. In addition, RCS models were used to show the exposure-response association between PM_2.5_ exposure and mortality risk stratified by dietary patter scores.

The joint effects of ambient PM_2.5_ exposure and dietary pattern scores in relation to mortality risk were evaluated using time-varying Cox regression models. Participants were categorized into six groups based on the combinations of PM_2.5_ exposure tertiles and dietary pattern scores dichotomized at the sex-specific median. Individuals with healthier dietary profiles (≥ median for DQI-I and MIND, < median for DII) and the first tertile of PM_2.5_ exposure served as the reference group.

In sensitivity analysis, to address potential confounding from pre-existing comorbidities and reverse causation, the stratified and joint analyses of PM_2.5_ exposure and dietary pattern scores in relation to mortality risk were repeated after excluding participants with history of CVD, cancer or COPD at baseline, deaths within two years after baseline, or those with less than two follow-up visits. Additionally, mortality follow-up was truncated in 2015 to align with PM_2.5_ exposure data availability. Furthermore, stratified analyses and joint effects of dietary patterns with baseline PM_2.5_ exposure on mortality risk were conducted.

Statistical analyses were conducted using STATA (version 18; StataCorp, College Station, TX, USA) and R (version 4.3.1; R Foundation for Statistical Computing, Vienna, Austria). Figures were generated using GraphPad Prism (version 9.5.0 for Windows; GraphPad Software, Boston, MA, USA). A two-sided *p*-value < 0.05 was considered statistically significant.

## Results

### Participant characteristics

The study included 3,937 community-living older adults, with women accounting for 50.01% of participants. The mean age at baseline was 72.47 (SD 5.19) years, and the average BMI was 23.68 (SD 3.30) kg/m^2^. A total of 1,856 all-cause deaths were observed over a median follow-up of 16.8 years, including 480 related to respiratory diseases and 403 from circulatory causes. Baseline characteristics are presented in Table 1. The mean annual PM_2.5_ concentration was 32.87 (SD 2.86) µg/m^3^ from 2000 to 2014.

**Table 1:** Baseline characteristics of participants

	All participants (*n* = 3,937)
Age, year	72.47 (5.19)
Sex, Female, %	50.01
Education, %	
No education	21.36
Primary or below	50.19
Secondary or above	28.45
Marital status, %	
Married	70.74
Widowed	24.69
Single/divorce/separated	4.57
Smoking status, %	
Non-smoker	63.35
Past smoker	29.74
Current smoker	6.91
Alcohol drinking, %	13.1
Number of chronic diseases, %	
0	15.21
1–2	53.24
≥ 3	31.55
Weight, kg	58.46 (9.78)
BMI, kg/m^2^	23.68 (3.30)
PASE score	91.19 (42.79)
Energy intake, kcal/day	1835.03 (574.51)
DQI-I	64.36 (9.58)
DII	−0.47 (1.48)
MIND	4.58 (0.94)

Mean (Standard Deviation) for continuous variables; percentage (%) for categorical variables

*Abbreviation*: *PM*_2.5_ fine particulate matter, *BMI* Body mass index, *PASE* Physical Activity Scale for the Elderly, *DQI-I* Diet Quality Index-International, *DII* Dietary inflammatory index, *MIND* The Mediterranean-DASH Intervention for Neurodegenerative Delay Diet score

### Association between long-term ambient PM_2.5_ exposure and mortality risk

Table 2 presents the associations between long-term ambient PM_2.5_ exposure and the risks of all-cause, respiratory, and circulatory mortality. The HRs for per 5 µg/m^3^ increase of PM_2.5_ exposure were 1.33 (95% CI: 1.23, 1.44) for all-cause mortality, 1.41 (95% CI: 1.21, 1.65) for respiratory mortality and 1.50 (95% CI: 1.27, 1.78) for circulatory mortality in the age- and sex-adjusted model. These associations did not change after adjusting for sociodemographic and lifestyle factors, with HRs for per 5 µg/m^3^ increase of PM_2.5_ exposure of 1.28 (95% CI: 1.18–1.39) for all-cause mortality, 1.31 (95% CI: 1.12, 1.54) for respiratory mortality and 1.48 (95% CI: 1.24, 1.76) for circulatory mortality. In addition, participants in the second and third tertile of PM_2.5_ exposure had significantly higher HRs of 1.31 (95% CI: 1.18, 1.47) and 1.30 (95% CI: 1.15, 1.47) for all-cause mortality, 1.41 (95% CI: 1.13, 1.76) and 1.41 (95% CI: 1.11, 1.80) for respiratory mortality, and 1.57 (95% CI: 1.22, 2.00) and 1.57 (95% CI: 1.20, 2.06) for circulatory mortality, compared to those in the first tertile. The overall exposure-response associations between PM_2.5_ concentrations and mortality outcomes, and the population distribution across the range of PM_2.5_ concentrations, are presented in Supplementary Figure S1. We observed significant non-linear associations for both all-cause and circulatory mortality (*p*-nonlinearity < 0.05). Specifically, the excess risks for these two outcomes increased with rising PM_2.5_ concentrations but appeared to plateau after PM_2.5_ concentrations exceeded approximately 35 µg/m^3^. In contrast, the association between PM_2.5_ exposure and respiratory mortality was approximately linear (*p*-nonlinearity = 0.0967).

**Table 2:** Associations between PM_2.5_ exposure and all-cause, respiratory, and circulatory morality

	Tertiles of PM_2.5_ exposure, HR (95% CI)			Per 5 µg/m^3^ increase of PM_2.5_ exposure
	T1	T2	T3	
All-cause mortality				
Model 1 ^a^	1.00 (Ref)	1.35 (1.21, 1.50)	1.37 (1.22, 1.54)	1.33 (1.23, 1.44)
Model 2 ^b^	1.00 (Ref)	1.31 (1.18, 1.47)	1.30 (1.15, 1.47)	1.28 (1.18, 1.39)
Respiratory mortality				
Model 1	1.00 (Ref)	1.50 (1.22, 1.85)	1.53 (1.21, 1.93)	1.41 (1.21, 1.65)
Model 2	1.00 (Ref)	1.41 (1.13, 1.76)	1.41 (1.11, 1.80)	1.31 (1.12, 1.54)
Circulatory mortality				
Model 1	1.00 (Ref)	1.54 (1.22, 1.95)	1.61 (1.25, 2.07)	1.50 (1.27, 1.78)
Model 2	1.00 (Ref)	1.57 (1.22, 2.00)	1.57 (1.20, 2.06)	1.48 (1.24, 1.76)

*Abbreviation*: *HR* Hazard ratio, *95% CI* 95% confidence interval, *Ref* Reference

^a^Model 1: adjusted for age (continuous) and sex (male, female)

^b^Model 2: adjusted for model 1 plus education level (no education; primary or below; secondary or above), marital status (married, widowed, single/separated/divorced), smoking status (never, former, current), current alcohol drinking (yes; no), number of chronic diseases (0; 1–2; ≥3 diseases), physical activity level (continuous), BMI categories (< 18.5; 18.5–22.9; 23–27.4; ≥27.5 kg/m^2^) and total energy intake (continuous)

### Stratified association between PM_2.5_ exposure and mortality risk by dietary pattern scores

**Fig. 1:**
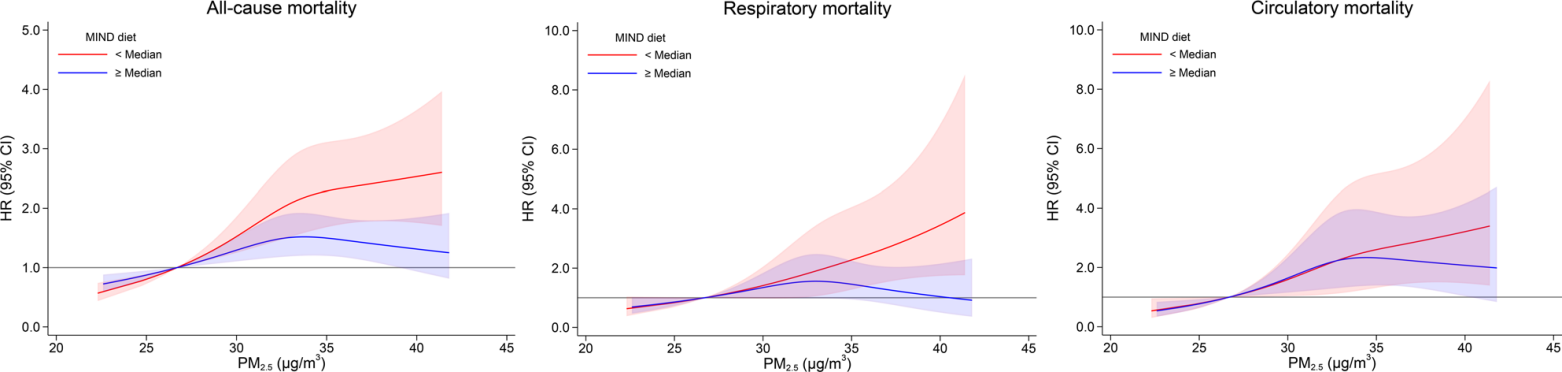
Stratified analysis of association between PM_2.5_ exposure and mortality risk by the MIND diet scores. In restricted cubic spline cox models, three knots were set at 10th, 50th, and 90th percentiles of PM2.5 concentrations, and the reference was 1st percentile. Confounders included age, sex, education level, marital status, smoking status, current alcohol drinking, number of chronic diseases, physical activity level, BMI categories and total energy intake

A significant interaction was observed between exposure to PM_2.5_ and the MIND diet in relation to all-cause mortality (*p*-interaction = 0.008), as shown in Table 3. Participants with lower MIND diet scores (< median) had higher all-cause mortality risk associated with increased PM_2.5_ concentrations, with HRs of 1.45 (95% CI: 1.27, 1.65). However, the positive association between PM_2.5_ exposure and the risk of all-cause mortality was attenuated among individuals with higher MIND scores (≥ median), with HRs of 1.18 (95% CI: 1.06, 1.32) for per 5 µg/m³ increase of PM_2.5_ exposure. Similarly, there was a significant interaction between exposure to PM_2.5_ and the MIND diet in relation to respiratory mortality (*p*-interaction = 0.022). No association was found between exposure to PM_2.5_ and respiratory mortality among those with higher MIND diet scores (HR: 1.14, 95% CI: 0.91, 1.41; per 5 µg/m^3^ increase of PM_2.5_ exposure), while the HR for respiratory mortality was 1.60 (95% CI: 1.24, 2.06) among those with lower MIND diet scores. However, there was no significant interaction in relation to circulatory mortality (*p*-interaction = 0.559). Besides, no significant interactions were observed between PM_2.5_ exposure with DQI-I or DII on all-cause, respiratory and circulatory mortality (all *p*-interaction > 0.05). Figure 1 shows the associations between long-term exposure to PM_2.5_ exposure and mortality stratified by MIND diet scores using restricted cubic spline models. The results were consistent with those in Table 3. Higher MIND diet scores (≥ median) attenuated the adverse associations between PM_2.5_ concentrations and both all-cause and respiratory mortality.

**Table 3:** Associations between PM_2.5_ exposure and all-cause, respiratory, and circulatory mortality stratified by dietary patterns

	Tertiles of PM_2.5_ exposure, HR (95% CI)			Per 5 µg/m^3^ increase of PM_2.5_ exposure	*p*-interaction
	T1	T2	T3		
All-cause mortality					
DQI-I					0.883
≥ Median	1.00 (Ref)	1.33 (1.13, 1.57)	1.32 (1.11, 1.57)	1.30 (1.15, 1.46)	
< Median	1.00 (Ref)	1.30 (1.12, 1.53)	1.30 (1.09, 1.55)	1.28 (1.14, 1.44)	
DII					0.974
≥ Median	1.00 (Ref)	1.27 (1.09, 1.48)	1.39 (1.17, 1.64)	1.27 (1.14, 1.43)	
< Median	1.00 (Ref)	1.36 (1.15, 1.61)	1.19 (0.99, 1.44)	1.29 (1.14, 1.46)	
MIND					0.008
≥ Median	1.00 (Ref)	1.21 (1.04, 1.39)	1.12 (0.95, 1.32)	1.18 (1.06, 1.32)	
< Median	1.00 (Ref)	1.53 (1.28, 1.84)	1.63 (1.34, 1.98)	1.45 (1.27, 1.65)	
Respiratory mortality					
DQI-I					0.861
≥ Median	1.00 (Ref)	1.35 (0.97, 1.87)	1.45 (1.02, 2.06)	1.39 (1.09, 1.76)	
< Median	1.00 (Ref)	1.46 (1.08, 1.98)	1.38 (0.98, 1.95)	1.27 (1.01, 1.59)	
DII					0.351
≥ Median	1.00 (Ref)	1.34 (0.99, 1.82)	1.32 (0.94, 1.86)	1.20 (0.95, 1.50)	
< Median	1.00 (Ref)	1.49 (1.08, 2.07)	1.50 (1.05, 2.13)	1.44 (1.13, 1.84)	
MIND					0.022
≥ Median	1.00 (Ref)	1.21 (0.91, 1.61)	1.14 (0.82, 1.58)	1.14 (0.91, 1.41)	
< Median	1.00 (Ref)	1.77 (1.23, 2.54)	1.96 (1.34, 2.87)	1.60 (1.24, 2.06)	
Circulatory mortality					
DQI-I					0.320
≥ Median	1.00 (Ref)	1.56 (1.10, 2.17)	1.55 (1.07, 2.23)	1.40 (1.10, 1.79)	
< Median	1.00 (Ref)	1.58 (1.10, 2.26)	1.62 (1.09, 2.40)	1.59 (1.22, 2.07)	
DII					0.920
≥ Median	1.00 (Ref)	1.65 (1.18, 2.31)	1.78 (1.24, 2.55)	1.49 (1.17, 1.88)	
< Median	1.00 (Ref)	1.46 (1.01, 2.12)	1.36 (0.91, 2.05)	1.46 (1.11, 1.93)	
MIND					0.559
≥ Median	1.00 (Ref)	1.53 (1.12, 2.10)	1.50 (1.06, 2.12)	1.42 (1.13, 1.79)	
< Median	1.00 (Ref)	1.63 (1.10, 2.43)	1.70 (1.11, 2.62)	1.57 (1.18, 2.08)	

*Abbreviation*: *PM*_2.5_ fine particulate matter, *HR* Hazard ratio, *95% CI* 95% confidence interval, *Ref* Reference, *DQI-I* Diet Quality Index-International, *DII* Dietary inflammatory index, *MIND* The Mediterranean-DASH Intervention for Neurodegenerative Delay Diet score

Adjusted for age (continuous), sex (male, female), education level (no education; primary or below; secondary or above), marital status (married, widowed, single/separated/divorced), smoking status (never, former, current), current alcohol drinking (yes; no), number of chronic diseases (0; 1–2; ≥3 diseases), physical activity level (continuous), BMI categories (< 18.5; 18.5–22.9; 23–27.4; ≥27.5 kg/m^2^) and total energy intake (continuous)

### Combined associations of PM_2.5_ exposure and dietary patterns on mortality risk

**Fig. 2:**
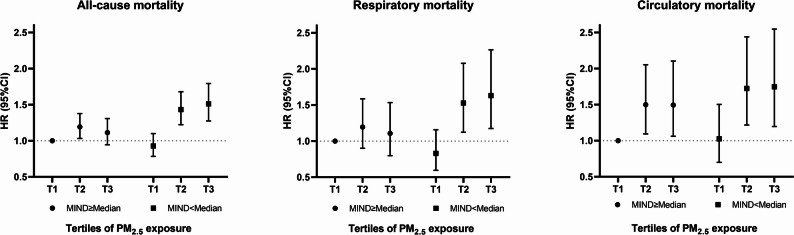
Joint associations of the MIND diet scores and tertiles of PM_2.5_ exposure on mortality risk. Confounders included age, sex, education level, marital status, smoking status, current alcohol drinking, number of chronic diseases, physical activity level, BMI categories and total energy intake

The combined associations of long-term PM_2.5_ exposure and MIND diet on mortality risk are shown in Fig. 2. The combination of higher MIND diet scores (≥ median) and the lowest tertile of PM_2.5_ exposure was the reference. Among individuals with higher MIND diet scores (≥ median), PM_2.5_ exposure was not significantly associated with increased risks of all-cause, respiratory, or circulatory mortality. Those in the third tertile of PM_2.5_ exposure with lower MIND diet scores (< median) had the highest HRs of 1.51 (95% CI: 1.27, 1.79) for all-cause mortality, 1.63 (95% CI: 1.17, 2.26) for respiratory mortality, and 1.75 (95% CI: 1.20, 2.55) for circulatory mortality.

### Sensitivity analysis

In sensitivity analyses, the stratified and joint associations between PM_2.5_ exposure and dietary patterns on all-cause, respiratory, and circulatory mortality remained consistent after excluding individuals with history of CVD, cancer, or COPD at baseline, excluding death within two years after baseline, excluding those with less than two follow-up visits or mortality follow-up truncated at 2015 (Supplementary Table S1 – Table S5). However, the interaction between exposure to PM_2.5_ and adherence to the MIND diet on respiratory mortality became borderline significant after excluding participants with fewer than two follow-up visits (*p*-interaction = 0.098) or when mortality follow-up period was truncated at 2015 (*p*-interaction = 0.076). The number of respiratory deaths may lack sufficient power to detect interactions in these sensitivity analyses. Although no significant interaction was observed, the associations between PM_2.5_ exposure and respiratory mortality were more pronounced among individuals with lower MIND diet scores (< median) compared with those with higher MIND diet scores (≥ median) in these sensitivity analyses (Supplementary Tables S3 and Table S4). Besides, substituting baseline PM_2.5_ exposure with long-term PM_2.5_ exposure did not change the results, with higher MIND diet scores (≥ median) mitigating PM_2.5_-associated all-cause and respiratory mortality risks (Supplementary Tables S6 and Table S7).

## Discussion

This prospective cohort study involving 3,937 older Chinese adults residing in the community observed positive associations between long-term ambient PM_2.5_ exposure and elevated risks of all-cause, respiratory and circulatory mortality. Among three dietary patterns examined (DQI-I, DII and MIND diet), we found that greater adherence to MIND diet may mitigate the adverse association between increased PM_2.5_ exposure and the risk of all-cause and respiratory mortality. In contrast, we did not observe a balanced diet (higher DQI-I scores) or an anti-inflammatory diet (lower DII scores) may attenuate these risks.

Among various air pollutants, PM_2.5_ has been identified as the main hazardous constituent [15]. Increasing evidence indicates that exposure to PM_2.5_ poses significant life-threatening risks [36, 37]. The overall average concentration of PM_2.5_ was 32.9 (SD: 2.9; median: 33.0, range: 22.3–41.8) µg/m^3^ among study participants in Hong Kong between 2000 and 2014, which is consistent with findings from the Chinese Elderly Health Service cohort in Hong Kong. That cohort reported a median PM_2.5_ concentration of 34.6 µg/m³ during 1998–2011, as assessed by a satellite-based model [38]. The PM_2.5_ in Hong Kong was much higher than the current WHO guideline annual exposure level of 10 µg/m^3^ for PM_2.5_ [39]. In addition, this level was higher than the PM_2.5_ concentrations in the UK biobank study (mean ± SD: 10.0 ± 1.1 µg/m^3^) [10] and in six U.S. states (mean ± SD: 12.9 ± 3.2 µg/m^3^, ranging from 3.4 to 23.0 µg/m^3^) [11], but lower than that reported in 22 provinces of mainland China from the Chinese Longitudinal Healthy Longevity Survey (mean ± SD: 49.3 ± 13.8 µg/m^3^, ranging from 9 to 106 µg/m^3^) [40]. Hong Kong ranks among the world’s most crowded urban areas and experiences a comparatively limited variation in PM_2.5_ levels [41]. We observed the robust associations between long-term PM_2.5_ exposure and the risks of all-cause, respiratory, and circulatory mortality among older adults in high-density urban areas, Hong Kong, after controlling demographics and lifestyle factors such as education levels, smoking status, alcohol consumption, physical activity, BMI, and other covariates.

The MIND diet may attenuate the elevated mortality risks linked to long-term ambient PM_2.5_ exposure. The MIND diet emphasizes higher intakes of green leafy vegetables, berries, whole grains, nuts, non-fried fish and limiting foods rich in saturated fats and animal origin, which was designed to promote brain health and was based on the DASH and Mediterranean diet [19]. In a prospective cohort of U.S. women, the inverse association between higher exposure to PM_2.5_ and lower white matter volume was more prominent among those with lower MIND diet adherence and became insignificant among those with greater MIND diet adherence [7]. Previous studies have indicated that higher vegetable intake, a plant-based diet and the Mediterranean diet may modify the association between PM_2.5_ exposure and health outcomes [9–11, 40]. Moreover, several short-term intervention studies have found that dietary supplements, including vitamin C, vitamin E, fish oil, olive oil, and B vitamins, can protect against the detrimental cardiopulmonary effects of air pollution [42, 43]. These dietary factors may help reduce oxidative stress and inflammatory damage from air pollution [43, 44]. In addition, a screening study has identified berries and nuts as among the highest sources of antioxidants in various foods [45]. This suggests that healthy diets enriched in both antioxidants and anti-inflammatory properties, such as the MIND diet, may counteract the adverse health effects of PM_2.5_ exposure.

This study found that the MIND diet modified the PM_2.5_ exposure-respiratory mortality association, but no significant interaction was observed for cardiovascular mortality. The respiratory system is the initial barrier to air pollution and is highly susceptible to PM_2.5_ exposure. Ambient particulate matter primarily targets the respiratory system, depositing in the lungs and generating reactive oxygen species (ROS) through redox cycling, or activating inflammatory pathways and lymphocytes, leading to pulmonary toxicity [46]. In addition, Type II alveolar cells may react sensitively to PM_2.5_-induced oxidative stress, causing an inflammatory response that does not rely on immune cells [47]. PM_2.5_ then crosses the alveolar-capillary barrier and enters the bloodstream, causing systemic oxidative stress and inflammation, and leading to circulatory and other diseases [48]. Therefore, the adverse impact of PM_2.5_ on the cardiovascular system is a cumulative chronic response, potentially involving many confounding factors. This may explain the lack of significant interaction between the MIND diet and PM_2.5_ exposure on cardiovascular mortality in this study. However, joint analyses suggested that following the MIND diet reduced the cardiovascular death risk linked to higher PM_2.5_ exposure (Fig. 2). Notably, individuals with following the MIND diet had a lower risk of circulatory mortality (Supplementary Table S8), but no significant association was observed in relation to respiratory mortality in this study. This finding was consistent with previous research indicating the cardiovascular benefits of following the MIND diet [49]. However, to our knowledge, the potential association between MIND diet adherence and respiratory mortality has yet to be explored.

Anti-inflammatory diets and high overall diet quality may not completely counter higher mortality risks related to PM_2.5_ exposure in this study. While DII incorporates certain antioxidant nutrients like vitamin C, vitamin E, and polyunsaturated fatty acids, its calculation primarily focuses on their anti-inflammatory effects rather than their antioxidant capacity [18]. Interestingly, a prospective study among 8,495 Chinese pregnant women showed that an anti-inflammatory diet may mitigate the impact of air pollution on the development of gestational diabetes mellitus [50]. This study used the empirical dietary inflammatory pattern (EDIP) score, which was based on 17 food groups and their inflammatory effects [50], differing from DII which mainly focuses on nutrient calculations [18]. EDIP and MIND diets may overlap by emphasizing the consumption of green leafy vegetables and reducing the consumption of red and processed meats to mitigate inflammation and promote health [51]. On the other hand, diet quality (as measured by DQI-I) focuses on the overall nutritional value of a diet [20]. Evidence from the UK Biobank indicated that high dietary diversity could potentially modify the effects of air pollution on the development, progression, and fatality of type 2 diabetes [8]. A possible explanation may be that high dietary diversity provides sufficient phytochemicals, bioactive components, vitamins, multiple minerals, and omega-3 polyunsaturated fatty acids, which may show antioxidative and anti-inflammatory effects [8]. The observed inconsistent results may stem from differences in how dietary diversity index and DQI-I calculate variety. Dietary diversity considers 8 animal-based and 10 plant-based foods [8], while the variety of DQI-I is based on the overall dietary variety across only 5 major food groups and the protein sources variety [20]. Although DQI-I–assessed diet quality offers potential health benefits, it may not be sufficient to fully counteract the detrimental effects of PM_2.5_ exposure. More studies are needed to explore how antioxidative and anti-inflammatory dietary patterns or a balanced and enriched diet influence the adverse health impact of air pollution exposure.

This prospective cohort study has several strengths, including the relatively large sample size of older adults, an extended follow-up period, the validated assessment to estimate dietary patterns, reliable prediction models of PM_2.5_ concentrations, and mortality data sourced from an official registry. In addition, the analysis accounted for a wide range of sociodemographic and lifestyle covariates in time-varying Cox models with several time points. However, there are several limitations in this study. First, indoor PM_2.5_ concentrations, participants’ behaviors such as opening windows, using air conditioning and indoor air purifiers, the time spent indoors and outdoors, ambient temperature and humidity could introduce confounding factors [52], but our analysis did not include information on these aspects. Second, changes in the residential addresses of participants during follow-up could influence the estimated PM_2.5_ concentrations, as they were calculated based on baseline addresses. At the 14-year follow-up, we collected data on the current address. Over 80% of participants had not moved from their baseline residential address. In addition, from the 14-year follow-up to the 18-year follow-up, 94.6% of participants did not change their address. Third, we did not analyze other air pollutants such as PM_2.5−10_, PM_10_, NO_2_ and NO_x_ in this study. Fourth, dietary patterns were assessed only at baseline. The potential effects of changes in dietary patterns over time could not be assessed. Although prior studies suggest that dietary behaviors among adults aged ≥ 55 years tended to remain relatively stable over a 4-year period [53], and that over half of older adults maintained consistent dietary habits over a 10-year follow-up [54], long-term shifts in eating habits cannot be entirely ruled out. Therefore, the possibility of misclassification of dietary exposure cannot be ruled out, and this limitation should be considered when interpreting the results. Fifth, our follow-up period ended in 2017. While ambient PM_2.5_ levels and compositions may have evolved in recent years, the fundamental biological mechanisms and exposure-response relationships between PM_2.5_ and health outcomes are unlikely to have changed. Therefore, these findings may remain applicable to current public health contexts. Finally, the study participants consisted of volunteers who generally had higher education attainments and greater health awareness than the general older adult population in Hong Kong, possibly resulting in an underestimation of the observed associations. In addition, the generalizability of the findings to other populations may be limited.

## Conclusion

Findings from this cohort study indicate that greater adherence to the MIND diet may offset the adverse mortality effects of PM_2.5_ exposure among older adults living in high-density urban areas. However, a balanced diet or an anti-inflammatory diet may be insufficient to mitigate these risks. The MIND diet, characterized by higher consumption of green leafy vegetables, whole grains, nuts, berries, and non-fried fish, alongside limited intake of animal-based and high saturated fat foods, may help counteract oxidative stress induced by PM_2.5_ exposure. The antioxidative dietary approach may effectively mitigate the health impacts of PM_2.5_ exposure at an individual level.

## Supplementary Information

Supplementary Material 1

## Data Availability

The datasets generated and/or analysed during the current study are not publicly available but are available from the corresponding author on reasonable request.

## Abbreviations

PM_2.5_: Fine particulate matter
DQI-I: Diet Quality Index-International
DII: Dietary Inflammatory Index
DASH: Dietary Approaches to Stop Hypertension
MIND: Mediterranean-DASH Intervention for Neurodegenerative Delay
HR: Hazard ratio
CI: Confidence interval
CVD: Cardiovascular disease
LUR: Land use regression
FFQ: Food frequency questionnaire
ICD-10: International Statistical Classification of Diseases and Related Health Problems, 10th Revision
COPD: Chronic obstructive lung disease
PASE: Physical Activity Scale of the Elderly
BMI: Body mass index
SD: Standard deviation
Ref: Reference
