# Supplementary material for: The potential role of dietary patterns in modifying the association between ambient PM2.5 exposure and mortality in elderly Hong Kong Chinese
Source: Environ Health. 2026 Apr 17;25:47. doi: 10.1186/s12940-026-01296-6 (PMC13224638; doi:10.1186/s12940-026-01296-6)
Supplement: Supplementary file 1 — Supplementary Material 1 [file 12940_2026_1296_MOESM1_ESM.docx]

**Supplementary materials**

**Title: The potential role of dietary patterns in modifying the association between ambient PM_2.5_ exposure and mortality in elderly Hong Kong Chinese**

**Author:** Shu-Yi Li ^1^, Jason Leung ^2^, Zhi-Hui Lu ^3^, Kin-Fai Ho ^4^, Yi Su ^5^, Blanche Yu ^2,6^, Timothy Kwok ^1,2,*^


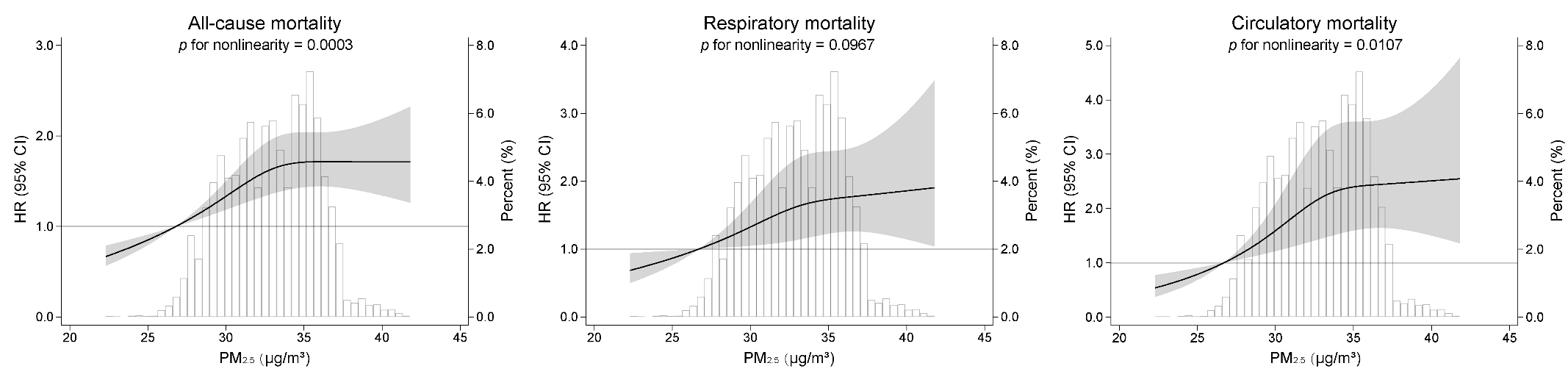


**Figure S1.** Overall exposure-response association between PM_2.5_ exposure and mortality risk and the population distribution of PM_2.5_ concentrations.

The restricted cubic spline (RCS) curves show the hazard ratios (95% confidence interval) for all-cause, respiratory, and circulatory mortality associated with PM_2.5_ exposure. In RCS cox models, three knots were set at 10^th^, 50^th^, and 90^th^ percentiles of PM_2.5_ concentrations, and the reference was 1^st^ percentile. Models were adjusted for age, sex, education level, marital status, smoking status, current alcohol drinking, number of chronic diseases, physical activity level, BMI categories and total energy intake. The histogram presents the population distribution (frequency) across the range of PM_2.5_ concentrations. The RCS curves and the histogram share the same X-axis of PM_2.5_ concentrations.

**Table S1.** Sensitivity analysis for associations between long-term PM_2.5_ exposure and all-cause, respiratory, and circulatory mortality stratified by dietary pattern scores (excluding those with CVD, cancer or COPD at baseline).

|  | Tertiles of PM_2.5_ exposure, HR (95% CI) | | | Per 5μg/m^3^ increment of PM_2.5_ concentrations | *p*-interaction |
| --- | --- | --- | --- | --- | --- |
|  | T1 | T2 | T3 |  |  |
| **All-cause mortality** | |  |  |  |  |
| DQI-I |  |  |  |  | 0.984 |
| ≥ Median | 1.00 (Ref) | 1.31 (1.11, 1.54) | 1.30 (1.08, 1.55) | 1.29 (1.14, 1.45) |  |
| < Median | 1.00 (Ref) | 1.27 (1.08, 1.50) | 1.28 (1.06, 1.54) | 1.26 (1.12, 1.43) |  |
| DII |  |  |  |  | 0.929 |
| ≥ Median | 1.00 (Ref) | 1.24 (1.05, 1.45) | 1.37 (1.15, 1.64) | 1.27 (1.13, 1.43) |  |
| < Median | 1.00 (Ref) | 1.33 (1.13, 1.58) | 1.18 (0.98, 1.43) | 1.27 (1.12, 1.44) |  |
| MIND |  |  |  |  | 0.007 |
| ≥ Median | 1.00 (Ref) | 1.18 (1.02, 1.37) | 1.10 (0.93, 1.30) | 1.17 (1.04, 1.30) |  |
| < Median | 1.00 (Ref) | 1.50 (1.24, 1.82) | 1.63 (1.33, 2.00) | 1.45 (1.26, 1.66) |  |
| **Respiratory mortality** | |  |  |  |  |
| DQI-I |  |  |  |  | 0.942 |
| ≥ Median | 1.00 (Ref) | 1.29 (0.93, 1.81) | 1.42 (0.99, 2.03) | 1.37 (1.07, 1.76) |  |
| < Median | 1.00 (Ref) | 1.44 (1.05, 1.97) | 1.34 (0.93, 1.92) | 1.24 (0.98, 1.56) |  |
| DII |  |  |  |  | 0.302 |
| ≥ Median | 1.00 (Ref) | 1.28 (0.93, 1.75) | 1.27 (0.89, 1.82) | 1.17 (0.92, 1.48) |  |
| < Median | 1.00 (Ref) | 1.50 (1.08, 2.09) | 1.47 (1.02, 2.12) | 1.44 (1.12, 1.84) |  |
| MIND |  |  |  |  | 0.027 |
| ≥ Median | 1.00 (Ref) | 1.20 (0.90, 1.61) | 1.10 (0.78, 1.54) | 1.12 (0.89, 1.40) |  |
| < Median | 1.00 (Ref) | 1.67 (1.15, 2.42) | 1.90 (1.28, 2.82) | 1.56 (1.20, 2.23) |  |
| **Circulatory mortality** | |  |  |  |  |
| DQI-I |  |  |  |  | 0.258 |
| ≥ Median | 1.00 (Ref) | 1.57 (1.11, 2.22) | 1.56 (1.07, 2.27) | 1.41 (1.09, 1.81) |  |
| < Median | 1.00 (Ref) | 1.59 (1.09, 2.33) | 1.71 (1.13, 2.60) | 1.66 (1.26, 2.18) |  |
| DII |  |  |  |  | 0.433 |
| ≥ Median | 1.00 (Ref) | 1.82 (1.27, 2.60) | 2.04 (1.39, 3.00) | 1.61 (1.26, 2.06) |  |
| < Median | 1.00 (Ref) | 1.36 (0.94, 1.90) | 1.27 (0.84, 1.93) | 1.39 (1.05, 1.84) |  |
| MIND |  |  |  |  | 0.458 |
| ≥ Median | 1.00 (Ref) | 1.50 (1.09, 2.09) | 1.51 (1.06, 2.16) | 1.44 (1.13, 1.82) |  |
| < Median | 1.00 (Ref) | 1.72 (1.13, 2.62) | 1.84 (1.17, 2.90) | 1.63 (1.22, 2.19) |  |

Abbreviation: PM_2.5_, fine particulate matter; HR, hazard ratio; 95%CI, 95% confidence interval; Ref, reference; DQI-I, Diet Quality Index-International; DII, dietary inflammatory index; MIND, the Mediterranean-DASH Intervention for Neurodegenerative Delay Diet score.

Excluding those with CVD (n=815), cancer (n=131) or COPD (n=247) at baseline. Adjusted for age, sex, education level, marital status, smoking status, current alcohol drinking, number of chronic diseases, physical activity level, BMI categories and total energy intake.

**Table S2.** Sensitivity analysis for associations between long-term PM_2.5_ exposure and all-cause, respiratory, and circulatory mortality stratified by dietary pattern scores (excluding death within two years after baseline).

|  | Tertiles of PM_2.5_ exposure, HR (95% CI) | | | Per 5μg/m^3^ increment of PM_2.5_ concentrations | *p*-interaction |
| --- | --- | --- | --- | --- | --- |
|  | T1 | T2 | T3 |  |  |
| **All-cause mortality** | |  |  |  |  |
| DQI-I |  |  |  |  | 0.912 |
| ≥ Median | 1.00 (Ref) | 1.33 (1.12, 1.56) | 1.35 (1.13, 1.61) | 1.31 (1.16, 1.48) |  |
| < Median | 1.00 (Ref) | 1.31 (1.11, 1.54) | 1.33 (1.11, 1.59) | 1.30 (1.15, 1.46) |  |
| DII |  |  |  |  | 0.822 |
| ≥ Median | 1.00 (Ref) | 1.24 (1.06, 1.45) | 1.40 (1.18, 1.66) | 1.28 (1.14, 1.44) |  |
| < Median | 1.00 (Ref) | 1.39 (1.18, 1.65) | 1.25 (1.04, 1.52) | 1.31 (1.16, 1.49) |  |
| MIND |  |  |  |  | 0.008 |
| ≥ Median | 1.00 (Ref) | 1.21 (1.05, 1.40) | 1.15 (0.98, 1.40) | 1.19 (1.07, 1.33) |  |
| < Median | 1.00 (Ref) | 1.51 (1.26, 1.83) | 1.67 (1.36, 2.03) | 1.46 (1.28, 1.67) |  |
| **Respiratory mortality** | |  |  |  |  |
| DQI-I |  |  |  |  | 0.827 |
| ≥ Median | 1.00 (Ref) | 1.35 (0.97, 1.87) | 1.45 (1.02, 2.06) | 1.39 (1.09, 1.77) |  |
| < Median | 1.00 (Ref) | 1.43 (1.05, 1.94) | 1.42 (1.01, 2.00) | 1.29 (1.03, 1.62) |  |
| DII |  |  |  |  | 0.330 |
| ≥ Median | 1.00 (Ref) | 1.31 (0.96, 1.78) | 1.33 (0.95, 1.88) | 1.21 (0.96, 1.52) |  |
| < Median | 1.00 (Ref) | 1.52 (1.10, 2.10) | 1.52 (1.06, 2.17) | 1.46 (1.15, 1.86) |  |
| MIND |  |  |  |  | 0.021 |
| ≥ Median | 1.00 (Ref) | 1.21 (0.91, 1.61) | 1.15 (0.83, 1.60) | 1.14 (0.92, 1.42) |  |
| < Median | 1.00 (Ref) | 1.72 (1.20, 2.47) | 1.97 (1.34, 2.89) | 1.61 (1.25, 2.08) |  |
| **Circulatory mortality** | |  |  |  |  |
| DQI-I |  |  |  |  | 0.417 |
| ≥ Median | 1.00 (Ref) | 1.61 (1.14, 2.27) | 1.65 (1.14, 2.39) | 1.44 (1.12, 1.84) |  |
| < Median | 1.00 (Ref) | 1.51 (1.05, 2.18) | 1.58 (1.06, 2.36) | 1.59 (1.22, 2.07) |  |
| DII |  |  |  |  | 0.963 |
| ≥ Median | 1.00 (Ref) | 1.60 (1.14, 2.24) | 1.76 (1.22, 2.53) | 1.49 (1.17, 1.88) |  |
| < Median | 1.00 (Ref) | 1.53 (1.05, 2.22) | 1.46 (0.97, 2.20) | 1.51 (1.14, 1.99) |  |
| MIND |  |  |  |  | 0.634 |
| ≥ Median | 1.00 (Ref) | 1.55 (1.12, 2.14) | 1.57 (1.11, 2.23) | 1.45 (1.15, 1.84) |  |
| < Median | 1.00 (Ref) | 1.61 (1.08, 2.39) | 1.68 (1.09, 2.58) | 1.55 (1.17, 2.06) |  |

Excluding death within two years after baseline, n=67. Adjusted for age, sex, education level, marital status, smoking status, current alcohol drinking, number of chronic diseases, physical activity level, BMI categories and total energy intake.

**Table S3.** Sensitivity analysis for associations between long-term PM_2.5_ exposure and all-cause, respiratory, and circulatory mortality stratified by dietary pattern scores (excluding less than two follow-up visits).

|  | Tertiles of PM_2.5_ exposure, HR (95% CI) | | | Per 5μg/m^3^ increment of PM_2.5_ concentrations | *p*-interaction |
| --- | --- | --- | --- | --- | --- |
|  | T1 | T2 | T3 |  |  |
| **All-cause mortality** | |  |  |  |  |
| DQI-I |  |  |  |  | 0.788 |
| ≥ Median | 1.00 (Ref) | 1.21 (1.00,1.47) | 1.42 (1.16, 1.74) | 1.32 (1.15, 1.52) |  |
| < Median | 1.00 (Ref) | 1.22 (1.00, 1.48) | 1.30 (1.04, 1.63) | 1.28 (1.11, 1.49) |  |
| DII |  |  |  |  | 0.649 |
| ≥ Median | 1.00 (Ref) | 1.17 (0.97, 1.42) | 1.51 (1.23, 1.86) | 1.34 (1.16, 1.54) |  |
| < Median | 1.00 (Ref) | 1.27 (1.05, 1.55) | 1.25 (1.00, 1.55) | 1.28 (1.10, 1.48) |  |
| MIND |  |  |  |  | 0.001 |
| ≥ Median | 1.00 (Ref) | 1.10 (0.92, 1.30) | 1.12 (0.93, 1.37) | 1.16 (1.02, 1.32) |  |
| < Median | 1.00 (Ref) | 1.50 (1.19, 1.89) | 1.93 (1.51, 2.47) | 1.59 (1.35, 1.88) |  |
| **Respiratory mortality** | |  |  |  |  |
| DQI-I |  |  |  |  | 0.694 |
| ≥ Median | 1.00 (Ref) | 1.27 (0.87, 1.84) | 1.45 (0.97, 2.16) | 1.38 (1.05, 1.82) |  |
| < Median | 1.00 (Ref) | 1.30 (0.90, 1.90) | 1.24 (0.80, 1.93) | 1.19 (0.90, 1.59) |  |
| DII |  |  |  |  | 0.743 |
| ≥ Median | 1.00 (Ref) | 1.25 (0.86, 1.82) | 1.38 (0.90, 2.13) | 1.25 (0.94, 1.67) |  |
| < Median | 1.00 (Ref) | 1.35 (0.93, 1.96) | 1.37 (0.91, 2.06) | 1.34 (1.02, 1.76) |  |
| MIND |  |  |  |  | 0.098 |
| ≥ Median | 1.00 (Ref) | 1.12 (0.80, 1.56) | 1.13 (0.77, 1.67) | 1.13 (0.87, 1.46) |  |
| < Median | 1.00 (Ref) | 1.56 (1.01, 2.39) | 1.81 (1.14, 2.87) | 1.52 (1.12, 2.08) |  |
| **Circulatory mortality** | |  |  |  |  |
| DQI-I |  |  |  |  | 0.281 |
| ≥ Median | 1.00 (Ref) | 1.72 (1.15, 2.57) | 1.70 (1.10, 2.64) | 1.39 (1.04, 1.87) |  |
| < Median | 1.00 (Ref) | 1.58 (1.01, 2.49) | 1.68 (1.01, 2.82) | 1.68 (1.20, 2.36) |  |
| DII |  |  |  |  | 0.528 |
| ≥ Median | 1.00 (Ref) | 1.80 (1.19, 2.72) | 2.17 (1.38, 3.42) | 1.64 (1.22, 2.20) |  |
| < Median | 1.00 (Ref) | 1.65 (1.06, 2.56) | 1.34 (0.81, 2.22) | 1.41 (1.02, 1.96) |  |
| MIND |  |  |  |  | 0.173 |
| ≥ Median | 1.00 (Ref) | 1.57 (1.08, 2.28) | 1.44 (0.94, 2.20) | 1.36 (1.03, 1.80) |  |
| < Median | 1.00 (Ref) | 1.99 (1.19, 3.32) | 2.40 (1.37, 4.18) | 1.88 (1.31, 2.71) |  |

Excluding less than two follow-up visits, n=765. Adjusted for age, sex, education level, marital status, smoking status, current alcohol drinking, number of chronic diseases, physical activity level, BMI categories and total energy intake.

**Table S4.** Sensitivity analysis for associations between long-term PM_2.5_ exposure and all-cause, respiratory, and circulatory mortality stratified by dietary pattern scores (Mortality follow-up until 2015).

|  | Tertiles of PM_2.5_ exposure, HR (95% CI) | | | Per 5μg/m^3^ increment of PM_2.5_ concentrations | *p*-interaction |
| --- | --- | --- | --- | --- | --- |
|  | T1 | T2 | T3 |  |  |
| **All-cause mortality** | |  |  |  |  |
| DQI-I |  |  |  |  | 0.191 |
| ≥ Median | 1.00 (Ref) | 1.38 (1.11, 1.71) | 1.33 (1.05, 1.68) | 1.33 (1.13, 1.56) |  |
| < Median | 1.00 (Ref) | 1.59 (1.29, 1.97) | 1.84 (1.45, 2.32) | 1.52 (1.31, 1.77) |  |
| DII |  |  |  |  | 0.698 |
| ≥ Median | 1.00 (Ref) | 1.55 (1.26, 1.91) | 1.79 (1.44, 2.23) | 1.44 (1.24, 1.66) |  |
| < Median | 1.00 (Ref) | 1.38 (1.10, 1.73) | 1.25 (0.97, 1.62) | 1.37 (1.15, 1.62) |  |
| MIND |  |  |  |  | 0.007 |
| ≥ Median | 1.00 (Ref) | 1.26 (1.03, 1.54) | 1.23 (0.99, 1.54) | 1.23 (1.06, 1.43) |  |
| < Median | 1.00 (Ref) | 1.87 (1.14, 2.38) | 2.11 (1.64, 2.72) | 1.69 (1.43, 1.99) |  |
| **Respiratory mortality** | |  |  |  |  |
| DQI-I |  |  |  |  | 0.363 |
| ≥ Median | 1.00 (Ref) | 1.39 (0.85, 2.28) | 1.32 (0.75, 2.31) | 1.40 (0.97, 2.03) |  |
| < Median | 1.00 (Ref) | 1.74 (1.10, 2.76) | 2.17 (1.32, 3.56) | 1.49 (1.06, 2.08) |  |
| DII |  |  |  |  | 0.193 |
| ≥ Median | 1.00 (Ref) | 1.56 (1.01, 2.39) | 1.69 (1.05, 2.72) | 1.25 (0.91, 1.72) |  |
| < Median | 1.00 (Ref) | 1.70 (1.00, 2.90) | 1.67 (0.93, 3.00) | 1.73 (1.16, 2.58) |  |
| MIND |  |  |  |  | 0.076 |
| ≥ Median | 1.00 (Ref) | 1.30 (0.84, 2.01) | 1.32 (0.79, 2.20) | 1.21 (0.86, 1.71) |  |
| < Median | 1.00 (Ref) | 2.12 (1.24, 3.62) | 2.42 (1.38, 4.25) | 1.75 (1.22, 2.51) |  |
| **Circulatory mortality** | |  |  |  |  |
| DQI-I |  |  |  |  | 0.091 |
| ≥ Median | 1.00 (Ref) | 1.38 (0.89, 2.13) | 1.39 (0.87, 2.23) | 1.29 (0.94, 1.77) |  |
| < Median | 1.00 (Ref) | 2.03 (1.22, 3.39) | 2.96 (1.75, 4.99) | 2.13 (1.51, 3.00) |  |
| DII |  |  |  |  | 0.142 |
| ≥ Median | 1.00 (Ref) | 2.08 (1.32, 3.29) | 2.72 (1.70, 4.36) | 1.86 (1.38, 2.50) |  |
| < Median | 1.00 (Ref) | 1.18 (0.72, 1.94) | 1.20 (0.70, 2.06) | 1.25 (0.86, 1.81) |  |
| MIND |  |  |  |  | 0.275 |
| ≥ Median | 1.00 (Ref) | 1.47 (0.95, 2.77) | 1.60 (1.00, 2.55) | 1.36 (0.99, 1.86) |  |
| < Median | 1.00 (Ref) | 1.86 (1.12, 3.09) | 2.42 (1.43, 4.09) | 1.94 (1.38, 2.73) |  |

There were 1,180 all-cause deaths, 256 respiratory deaths and 268 circulatory deaths among 3,837 participants during a median of 12.56 years follow-up. Adjusted for age, sex, education level, marital status, smoking status, current alcohol drinking, number of chronic diseases, physical activity level, BMI categories and total energy intake.

**Table S5.** Sensitivity analysis for joint associations between tertiles of long term PM_2.5_ exposure and the MIND diet scores on all-cause, respiratory, and circulatory mortality.

|  | Tertiles of PM_2.5_ exposure, HR (95% CI) | | |
| --- | --- | --- | --- |
|  | T1 | T2 | T1 |
| **Excluding those with CVD, cancer or COPD at baseline** | | | |
| All-cause mortality |  |  |  |
| MIND≥ Median | 1.00 (Ref) | 1.17 (1.01, 1.36) | 1.09 (0.92, 1.29) |
| MIND < Median | 0.92 (0.77, 1.09) | 1.39 (1.18, 1.64) | 1.49 (1.25, 1.78) |
| Respiratory mortality |  |  |  |
| MIND≥ Median | 1.00 (Ref) | 1.19 (0.89, 1.59) | 1.08 (0.77, 1.51) |
| MIND < Median | 0.83 (0.59, 1.17) | 1.47 (1.07, 2.02) | 1.61 (1.14, 2.27) |
| Circulatory mortality |  |  |  |
| MIND ≥ Median | 1.00 (Ref) | 1.47 (1.06, 2.04) | 1.49 (1.05, 2.13) |
| MIND < Median | 0.97 (0.65, 1.44) | 1.73 (1.21, 2.48) | 1.79 (1.21, 2.65) |
| **Excluding death within two years after baseline** | | |  |
| All-cause mortality |  |  |  |
| MIND ≥ Median | 1.00 (Ref) | 1.19 (1.03, 1.38) | 1.14 (0.96, 1.34) |
| MIND < Median | 0.94 (0.79, 1.11) | 1.43 (1.22, 1.69) | 1.57 (1.32, 1.87) |
| Respiratory mortality |  |  |  |
| MIND ≥ Median | 1.00 (Ref) | 1.20 (0.90, 1.59) | 1.12 (0.81, 1.56) |
| MIND < Median | 0.84 (0.60, 1.17) | 1.51 (1.11, 2.06) | 1.67 (1.20, 2.32) |
| Circulatory mortality |  |  |  |
| MIND ≥ Median | 1.00 (Ref) | 1.51 (1.09, 2.08) | 1.55 (1.10, 2.20) |
| MIND < Median | 1.07 (0.73, 1.58) | 1.78 (1.25, 2.54) | 1.82 (1.24, 2.68) |
| **Excluding less than two follow-up visits** | |  |  |
| All-cause mortality |  |  |  |
| MIND ≥ Median | 1.00 (Ref) | 1.09 (0.92, 1.29) | 1.11 (0.92, 1.35) |
| MIND < Median | 0.84 (0.69. 1.03) | 1.29 (1.06, 1.56) | 1.61 (1.30, 1.98) |
| Respiratory mortality |  |  |  |
| MIND ≥ Median | 1.00 (Ref) | 1.12 (0.80, 1.56) | 1.12 (0.76, 1.65) |
| MIND < Median | 0.86 (0.59, 1.25) | 1.44 (0.99, 2.09) | 1.57 (1.04, 2.37) |
| Circulatory mortality |  |  |  |
| MIND ≥ Median | 1.00 (Ref) | 1.54 (1.06, 2.23) | 1.41 (0.93, 2.15) |
| MIND < Median | 0.85 (0.52, 1.37) | 1.76 (1.15, 2.68) | 1.97 (1.23, 3.13) |
| **Mortality follow-up until 2015** | | |  |
| All-cause mortality |  |  |  |
| MIND ≥ Median | 1.00 (Ref) | 1.26 (1.03, 1.53) | 1.24 (0.99, 1.54) |
| MIND < Median | 0.87 (0.68, 1.10) | 1.60 (1.30, 1.97) | 1.77 (1.43, 2.20) |
| Respiratory mortality |  |  |  |
| MIND ≥ Median | 1.00 (Ref) | 1.31 (0.85, 2.00) | 1.25 (0.76, 2.08) |
| MIND < Median | 0.67 (0.39, 1.13) | 1.41 (0.90, 2.20) | 1.70 (1.06, 2.72) |
| Circulatory mortality |  |  |  |
| MIND ≥ Median | 1.00 (Ref) | 1.48 (0.96, 2.29) | 1.70 (1.07, 2.70) |
| MIND < Median | 1.04 (0.62, 1.75) | 1.90 (1.21, 2.99) | 2.33 (1.47, 3.71) |

**Table S6.** Associations between baseline PM_2.5_ exposure and all-cause, respiratory, and circulatory mortality stratified by dietary pattern scores.

|  | Tertiles of baseline PM_2.5_ exposure,  HR (95% CI) | | | Per 5μg/m^3^ increment of PM_2.5_ concentrations | *p*-interaction |
| --- | --- | --- | --- | --- | --- |
|  | T1 | T2 | T3 |  |  |
| **All-cause mortality** | |  |  |  |  |
| DQI-I |  |  |  |  | 0.576 |
| ≥ Median | 1.00 (Ref) | 1.13 (0.96, 1.33) | 1.09 (0.92, 1.28) | 1.07 (0.94, 1.21) |  |
| < Median | 1.00 (Ref) | 1.05 (0.89, 1.23) | 1.04 (0.89, 1.22) | 1.01 (0.90, 1.14) |  |
| DII |  |  |  |  | 0.854 |
| ≥ Median | 1.00 (Ref) | 1.05 (0.90, 1.23) | 1.10 (0.94, 1.28) | 1.06 (0.94, 1.19) |  |
| < Median | 1.00 (Ref) | 1.08 (0.92, 1.28) | 1.01 (0.86, 1.20) | 1.01 (0.89, 1.15) |  |
| MIND |  |  |  |  | 0.027 |
| ≥ Median | 1.00 (Ref) | 0.99 (0.86, 1.16) | 0.92 (0.80, 1.07) | 0.95 (0.85, 1.06) |  |
| < Median | 1.00 (Ref) | 1.18 (0.99, 1.42) | 1.30 (1.09, 1.55) | 1.18 (1.03, 1.34) |  |
| **Respiratory mortality** | |  |  |  |  |
| DQI-I |  |  |  |  | 0.336 |
| ≥ Median | 1.00 (Ref) | 0.86 (0.61, 1.22) | 1.26 (0.92, 1.72) | 1.21 (0.95, 1.55) |  |
| < Median | 1.00 (Ref) | 1.23 (0.91, 1.66) | 1.01 (0.74, 1.38) | 0.98 (0.78, 1.23) |  |
| DII |  |  |  |  | 0.643 |
| ≥ Median | 1.00 (Ref) | 1.00 (0.73, 1.36) | 1.13 (0.83, 1.52) | 1.06 (0.84, 1.33) |  |
| < Median | 1.00 (Ref) | 1.15 (0.82, 1.61) | 1.13 (0.82, 1.56) | 1.12 (0.88, 1.43) |  |
| MIND |  |  |  |  | 0.039 |
| ≥ Median | 1.00 (Ref) | 0.88 (0.65, 1.18) | 0.91 (0.68, 1.21) | 0.93 (0.75, 1.16) |  |
| < Median | 1.00 (Ref) | 1.39 (0.97, 1.99) | 1.63 (1.15, 2.31) | 1.38 (1.06, 1.79) |  |
| **Circulatory mortality** | |  |  |  |  |
| DQI-I |  |  |  |  | 0.532 |
| ≥ Median | 1.00 (Ref) | 1.34 (0.96, 1.88) | 1.13 (0.80, 1.60) | 1.07 (0.82, 1.38) |  |
| < Median | 1.00 (Ref) | 1.12 (0.78, 1.61) | 1.27 (0.89, 1.81) | 1.20 (0.91, 1.57) |  |
| DII |  |  |  |  | 0.371 |
| ≥ Median | 1.00 (Ref) | 1.12 (0.81, 1.56) | 1.15 (0.83, 1.59) | 1.06 (0.82, 1.35) |  |
| < Median | 1.00 (Ref) | 1.38 (0.95, 2.01) | 1.25 (0.86, 1.82) | 1.23 (0.92, 1.63) |  |
| MIND |  |  |  |  | 0.511 |
| ≥ Median | 1.00 (Ref) | 1.18 (0.86, 1.62) | 1.06 (0.77, 1.45) | 1.06 (0.83, 1.35) |  |
| < Median | 1.00 (Ref) | 1.26 (0.85, 1.87) | 1.41 (0.95, 2.08) | 1.22 (0.91, 1.64) |  |

Adjusted for age, sex, education level, marital status, smoking, alcohol drinking, number of chronic diseases, physical activity, BMI and energy intake.

**Table S7.** Joint associations between baseline PM_2.5_ exposure and MIND diet on all-cause, respiratory, and circulatory mortality.

|  | Tertiles of baseline PM_2.5_ exposure, HR (95% CI) | | |
| --- | --- | --- | --- |
|  | T1 | T2 | T1 |
| **All-cause mortality** |  |  |  |
| MIND≥ Median | 1.00 (Ref) | 1.01 (0.87, 1.16) | 0.93 (0.81, 1.08) |
| MIND < Median | 0.94 (0.79, 1.11) | 1.12 (0.95, 1.32) | 1.21 (1.03, 1.41) |
| **Respiratory mortality** |  |  |  |
| MIND≥ Median | 1.00 (Ref) | 0.91 (0.68, 1.21) | 0.92 (0.69, 1.22) |
| MIND < Median | 0.80 (0.57, 1.11) | 1.08 (0.78, 1.48) | 1.24 (0.91, 1.68) |
| **Circulatory mortality** |  |  |  |
| MIND ≥ Median | 1.00 (Ref) | 1.19 (0.87, 1.62) | 1.07 (0.78, 1.47) |
| MIND < Median | 0.95 (0.65, 1.38) | 1.22 (0.85, 1.73) | 1.33 (0.94, 1.88) |

Adjusted for age, sex, education level, marital status, smoking, alcohol drinking, number of chronic diseases, physical activity, BMI and energy intake.

**Table S8.** Associations between dietary pattern scores and all-cause, respiratory, and circulatory mortality.

|  | **Mortality, HR (95% CI)** | | |
| --- | --- | --- | --- |
|  | **Total** | **Men** | **Women** |
| **DQI-I, per 10 units** |  |  |  |
| All-cause mortality |  |  |  |
| Model 1 ^a^ | 0.85 (0.82-0.90) | 0.86 (0.81-0.91) | 0.85 (0.79-0.92) |
| Model 2 ^b^ | 0.90 (0.85-0.94) | 0.91 (0.85-0.97) | 0.87 (0.80-0.94) |
| Respiratory mortality |  |  |  |
| Model 1 | 0.82 (0.75-0.90) | 0.85 (0.76-0.95) | 0.79 (0.67-0.92) |
| Model 2 | 0.89 (0.81-0.97) | 0.92 (0.81-1.03) | 0.82 (0.69-0.96) |
| Circulatory mortality |  |  |  |
| Model 1 | 0.90 (0.81-0.99) | 0.91 (0.80-1.04) | 0.87 (0.75-1.02) |
| Model 2 | 0.93 (0.83-1.03) | 0.93 (0.81-1.07) | 0.91 (0.76-1.07) |
| **DII, per 1 unit** |  |  |  |
| All-cause mortality |  |  |  |
| Model 1 | 1.11 (1.07-1.14) | 1.15 (1.10-1.20) | 1.06 (1.01-1.11) |
| Model 2 | 1.07 (1.03-1.11) | 1.10 (1.04-1.15) | 1.04 (0.98-1.10) |
| Respiratory mortality |  |  |  |
| Model 1 | 1.14 (1.07-1.21) | 1.14 (1.06-1.24) | 1.12 (1.02-1.24) |
| Model 2 | 1.07 (0.99-1.15) | 1.06 (0.96-1.16) | 1.09 (0.97-1.23) |
| Circulatory mortality |  |  |  |
| Model 1 | 1.12 (1.04-1.20) | 1.16 (1.06-1.28) | 1.07 (0.97-1.18) |
| Model 2 | 1.09 (1.00-1.18) | 1.15 (1.03-1.28) | 1.03 (0.91-1.16) |
| **MIND, per 1 unit** |  |  |  |
| All-cause mortality |  |  |  |
| Model 1 | 0.88 (0.84-0.93) | 0.88 (0.83-0.94) | 0.89 (0.83-0.97) |
| Model 2 | 0.90 (0.86-0.95) | 0.94 (0.88-1.01) | 0.91 (0.83-0.98) |
| Respiratory mortality |  |  |  |
| Model 1 | 0.88 (0.80-0.97) | 0.87 (0.77-0.97) | 0.94 (0.80-1.12) |
| Model 2 | 0.93 (0.84-1.02) | 0.96 (0.85-1.09) | 0.96 (0.81-1.15) |
| Circulatory mortality |  |  |  |
| Model 1 | 0.85 (0.77-0.95) | 0.87 (0.77-0.99) | 0.82 (0.70-0.97) |
| Model 2 | 0.86 (0.77-0.95) | 0.90 (0.78-1.03) | 0.84 (0.71-1.00) |

^a^ Model 1: adjusted for age and sex.

^b^ Model 2: adjusted for model 1 plus age, sex, education level, marital status, smoking, alcohol drinking, number of chronic diseases, physical activity, BMI and energy intake.
